# Window-Modulated Compounding Nakagami Parameter Ratio Approach for Assessing Muscle Perfusion with Contrast-Enhanced Ultrasound Imaging

**DOI:** 10.3390/s20123584

**Published:** 2020-06-24

**Authors:** Huang-Chen Lin, Shyh-Hau Wang

**Affiliations:** 1 Department of Computer Science and Information Engineering, Institute of Medical Informatics, National Cheng Kung University, No. 1, University Road, East District, Tainan City 70101, Taiwan; lukaslin886@gmail.com; 2 Intelligent Manufacturing Research Center, National Cheng Kung University, No. 1, University Road, East District, Tainan City 70101, Taiwan

**Keywords:** contrast-enhanced ultrasound, muscle perfusion, Nakagami parameter, compounding Nakagami imaging

## Abstract

The assessment of microvascular perfusion is essential for the diagnosis of a specific muscle disease. In comparison with the current available medical modalities, the contrast-enhanced ultrasound imaging is the simplest and fastest means for probing the tissue perfusion. Specifically, the perfusion parameters estimated from the ultrasound time-intensity curve (TIC) and statistics-based time–Nakagami parameter curve (TNC) approaches were found able to quantify the perfusion. However, due to insufficient tolerance on tissue clutters and subresolvable effects, these approaches remain short of reproducibility and robustness. Consequently, the window-modulated compounding (WMC) Nakagami parameter ratio imaging was proposed to alleviate these effects, by taking the ratio of WMC Nakagami parameters corresponding to the incidence of two different acoustic pressures from an employed transducer. The time–Nakagami parameter ratio curve (TNRC) approach was also developed to estimate perfusion parameters. Measurements for the assessment of muscle perfusion were performed from the flow phantom and animal subjects administrated with a bolus of ultrasound contrast agents. The TNRC approach demonstrated better sensitivity and tolerance of tissue clutters than those of TIC and TNC. The fusion image with the WMC Nakagami parameter ratio and B-mode images indicated that both the tissue structures and perfusion properties of ultrasound contrast agents may be better discerned.

## 1. Introduction

As the largest of the soft tissues, muscle is mainly functioning for the protection and composition of almost all essential organs in the human body. In response to a variety of external attacks, most of the muscle diseases are still difficult to be fully differentiated, since muscles belong to heterogeneous tissues [1]. Consequently, only a few muscle symptoms may be diagnosed with a specific myopathy that is predominantly associated with proximal muscle weakness [2,3]. To date, several medical imaging modalities have been developed that are capable of noninvasively detecting the compositions and variations of muscles, including the tissue edema, fatty, and atrophic changes [3,4]. Nevertheless, it remains difficult for the majority of medical imaging modalities to precisely diagnose a specific muscular disease, since the morphological changes of tissues are usually non-disease-specific. Therefore, it certainly is desirable to further explore and develop alternate diagnostics for better assessing the states of muscular disease or treatment effect covering the microvascular changes in a local muscle tissue.

Among current available medical imaging modalities, nuclear medicine imaging [5], positron emission tomography [6,7], and contrast-enhanced magnetic resonance imaging [8,9] have been utilized to detect and assess the microcirculation of muscle tissues. For example, the positron emission tomography incorporated with scintigraphy methods has been applied to detect the local tissue perfusion by tracing the injected radioactive agents in the bloodstream. However, positron emission tomography has not been routinely implemented in clinical diagnosis for assessing muscle perfusion, owing to several such considerations as the availability of scanners and radiopharmaceuticals, exposure of ionizing radiation, and insufficient spatial resolution of images. The contrast-enhanced magnetic resonance imaging is without the hazard of ionizing radiation and that has been demonstrated to be able to detect tissue perfusion [10]. It, however, is still encountering insufficient imaging sampling rate to acquire changes of the tissue perfusion thoroughly. In contrast to those just-mentioned modalities, ultrasound imaging is a relatively safe modality that is frequently employed to diagnose the tissue structures [3,11], movement [12], and blood flow [10,13]. The frequency of most of the diagnostic ultrasounds is less than 10 MHz, and that tends to result in a short-of-sufficient spatial resolution to measure the blood flow or perfusion in the capillary beds. Certainly, it is straightforward to increase the resolution of the ultrasound image by the increase of employed ultrasound frequency. For instance, a 50 MHz high-frequency ultrasound is with micrometer resolution capable of assessing the burn degree of wounded muscle tissue [14]. Nevertheless, the increase of ultrasound frequency tends to unavoidably increase the acoustic attenuation and then decrease the depth and contrast of image greatly [15,16].

To further alleviate these hurdles, the air-filled microbubbles were developed as the ultrasound contrast agents (UCAs); they are able to generate much more scattering energy than those of rigid spheres of the same size and to stably flow throughout the capillary vessels [17,18]. The administrated UCAs in the bloodstream allow the acquisition of contrast-enhanced ultrasound (CEUS) imaging over a certain duration, to estimate the tissue perfusion, as well as to estimate perfusion parameters by utilizing the time-intensity curve (TIC) [13,19] technique, similarly to that of contrast-enhanced magnetic resonance imaging. The CEUS imaging and TIC have been shown to be able to detect and localize an altered microcirculation for the diagnosis and treatment monitoring of such muscular diseases as myocardial infarction and ischemia [20], renal diseases [21], and advanced diabetes mellitus [22]. The impaired skeletal muscle perfusion of the lower legs was also found to be a hallmark of various diseases, microvascular involvement in diabetes mellitus [23,24], and compartment syndrome [10,25]. Furthermore, the perfusion parameters estimated from the corresponding TIC, typically including the arrival time (AT), time-to-peak (TTP), peak value (MAX), blood flow velocity (BFV), and area under the curve (AUC), have been applied to assess the muscle microcirculation [13,19]. These perfusion parameters have been applied to assess patients with peripheral arterial occlusive disease (PAOD) [22]. After engaging in exercise, the PAOD patients tended to have a significantly longer TTP than those of the control subjects. The absolute flow reserve and TIC perfusion parameters were found capable of better assessing the state and severity of impaired and diseased muscles. Moreover, patients with advanced stages of peripheral arterial disease resulted in a longer TTP, lower MAXs, and slower BFVs [23,24]. In general, TTP, among other perfusion parameters estimated from CEUS imaging, was reported to be a more reproducible and suitable parameter for clinically assessing the muscle perfusion [26,27]. However, the accuracy of ultrasound-intensity-based TIC approach with the CEUS imaging for estimating the microcirculation may still be affected by the variations of microbubble scattering corresponding to ultrasound scanner settings (such as gain, time–gain compensation, and dynamic range) and acoustic properties of tissues (such as attenuation and clutter signals associated with non-perfused area of tissues, and vessel walls) [28,29].

In comparison with conventional ultrasound-intensity-based TIC approach, the statistical analysis of ultrasound backscattering signals, using Nakagami statistics, has been validated to be capable of further reducing the effects of tissue clutter and attenuation [30]. Specifically, the Nakagami parameter is a shape parameter that is able to quantitatively differentiate the probability density function (PDF) of ultrasonic backscattered envelopes into Rayleigh, pre-Rayleigh, or post-Rayleigh distributions [30,31]. Subsequently, instead of ultrasound intensity, the Nakagami parameter was implemented into the time–Nakagami parameter curve (TNC) for estimating the flow velocities, ranging from 2 to 18 mm/s, from the flow conduit of a tissue-mimicking phantom with a bolus of UCAs administration [32,33]. The TNC approach has achieved consistent results to those of the TIC approach, and it has further demonstrated a better tolerance of tissue clutter signals in the region of interest (ROI), without the need to utilize an additional wall filter. However, the TNC approach may still be encountered with the subresolvable effect [32] corresponding to the condition as that of the tissue clutter signals are sufficiently larger than the perfused UCAs signals. The subresolvable effect may lead to the underestimated Nakagami parameters and also affect the reproducibility of perfusion parameters derived from the TNC approach [32]. Moreover, the TNC approach with CEUS imaging might also be affected by the ultrasound spectral and energy characteristics associated with the variations of transmitted ultrasound (such as pressure amplitude, frequency, phase, pulse length, and pulse repetition rate), as well as UCAs properties (including shell viscoelasticity and gas solubility) and behaviors (including oscillation, coalesce, jet, or fragment) [17,18]. Therefore, prior the estimation of perfusion parameters with TNC approach to be regularly applied in clinical applications, the sensitivity and uniformity of this approach associated with the influence of UCAs behaviors, tissue clutters, and measurement conditions are essential to be further investigated.

In the present study, extensive efforts for better assessing the muscle perfusion were made by proposing the window-modulated compounding (WMC) Nakagami parameter ratio imaging with CEUS imaging, to alleviate the effects of tissue clutter, UCAs behaviors, and ultrasound system factors. The WMC Nakagami ratio imaging was realized by taking the ratio of Nakagami parameters from signals of the same region of interest insonified by two different acoustic pressures alternatively. The measurements from flow phantom and animal subjects were arranged and carried out to verify the proposed approach. Subsequently, the time–Nakagami parameter ratio curve (TNRC) implemented from a sequence of backscattering signals corresponding to a constant flow and bolus injection of UCAs in the flow phantom and animal bloodstream, respectively, was acquired and utilized for perfusion parameters’ estimation. The WMC Nakagami parameter ratio images were subsequently fused with corresponding B-mode images, to better visualize and assess the perfusion areas of UCAs and the surrounding tissues.

## 2. Materials and Methods

### 2.1. Ultrasound Imaging System

**Figure 1:**
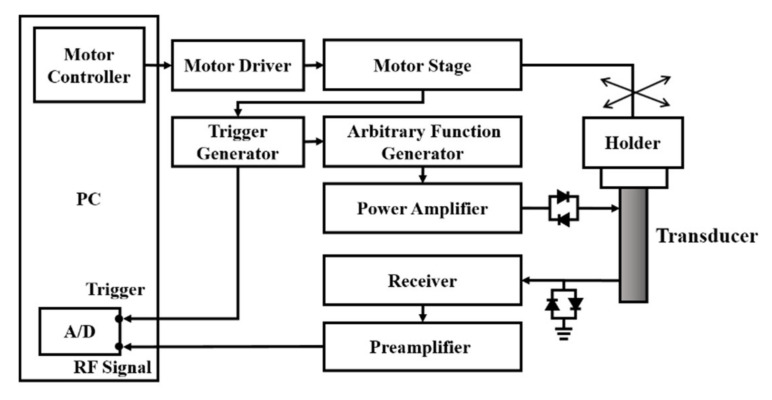
Schematic diagram of the ultrasound imaging system.

**Figure 2:**
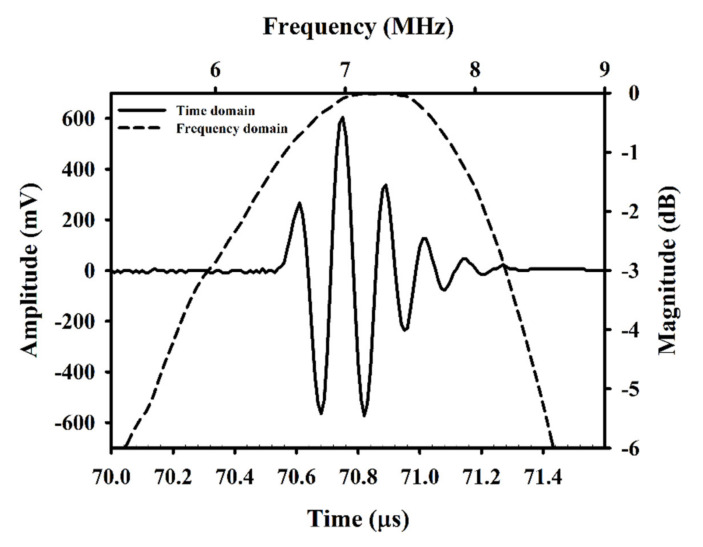
Pulse-echo response of the applied 7.5 MHz transducer.

The ultrasound imaging system for the present study was arranged and is schematically shown in Figure 1. The system mainly comprises a 7.5 MHz single-element ultrasound transducer (Panametrics V321, Waltham, MA, USA), pulser/receiver (Model 5072PR, Panametrics, Waltham, MA, USA), stage positioner (Model SGSP26-200, Sigma Koki, Tokyo, Japan) and controller (Model CSG-602R, Sigma Koki, Tokyo, Japan), and an 8-bit analog-to-digital converter (PXI-5152, National Instruments, Austin, TX, USA). The data-acquisition system was synchronized with the motor stage and controlled by the program developed with LabVIEW software (National Instruments, Austin, TX, USA). The pulse-echo response and characteristics of the transducer are given in Figure 2 and Table 1, respectively. The ultrasound transducer was driven by the bipolar signals generated and amplified, respectively, by an arbitrary function generator (AFG3252, Tektronix, Beaverton, OR, USA) and a broadband power amplifier (325LA, E&I, Rochester, NY, USA). Two acoustic pressures of 2.86 and 4.37 MPa, transmitted by the transducer and calibrated by a needle hydrophone (HNP-0200, ONDA Corporation, Sunnyvale, CA, USA), were prepared for flow phantom and animal experiments. The ultrasound signals were acquired and digitized at a 100 MHz sampling frequency. Subsequently, ultrasound B-mode images with a 42 dB dynamic range were formed, following a sequence of such processes as the filtering, Hilbert transform, logarithmic compression, and grayscale mapping. As a result, the imaging system achieves the axial and lateral image resolutions of 460 and 580 μm, respectively, measured from a 10 μm tungsten wire.

### 2.2. Flow Phantom Experiments

**Figure 3:**
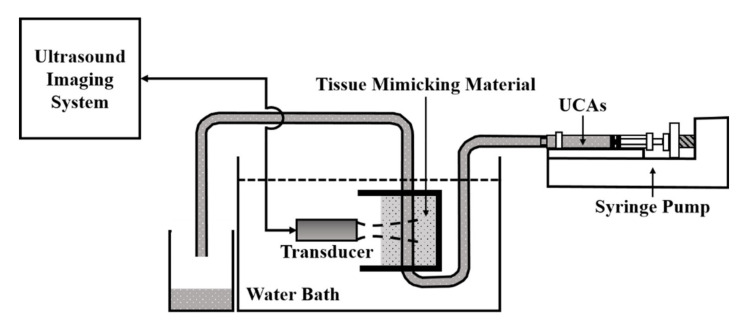
The arrangement for flow phantom experiments.

The arrangement of flow phantom experiments includes an ultrasound imaging system, flow phantom, and a syringe pump (KDS100, KD Scientific, New Hope, PA, USA), as given in Figure 3. The flow phantom is composed of a 1.5 × 1.5 mm^2^ flow conduit embedded in a tissue-mimicking material that was made of a mixture of gelatin, graphite powder, and deionized distilled water (DD water). A total of four concentrations of UCAs (USphere™, Trust Bio-Sonics, Hsin-Chu, Taiwan) were prepared by mixing a 50 μL of 2.5 × 10^10^ UCAs/mL with 25, 50, 100, and 200 mL Ringer’s solution. The mean diameter of UCAs, 0.6 μm approximately, was measured by a dynamic light-scattering system (Delsa™ Nano C, Beckman Coulter, Brea, CA, USA). The UCAs suspensions of various concentrations were continuously infused into the flow phantom, using a syringe pump, at an infusion rate of 140 mL/h or mean flow velocity of 4.75 mm/s. For each measurement, a sequence of 500 ultrasound images was acquired at a rate of 4 frames/s for 125 s. Each frame of 10 × 15 mm^2^ image is composed of 150 A-lines signals with a 100 μm scanning interval. The perfusion of UCAs was estimated from signals within a 2.5 × 4 mm^2^ ROI. A total of ten repeated experiments for each concentration of UCAs was measured.

### 2.3. Animal Experiments

**Figure 4:**
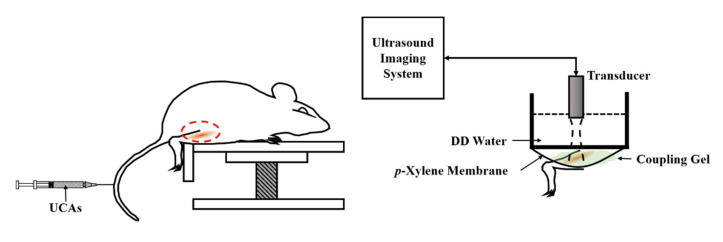
The arrangement for in vivo animal experiments.

In vivo animal experiments were subsequently performed from 20 male Sprague Dawley rats (10 weeks old and 433.3 ± 43.1 (mean ± standard deviation) g weight). The rats were taken care of in a typical rodent vivarium in which a fixed temperature and a 12 h light/dark cycle environment were equipped. For each animal experiment, the rat was anesthetized, and then the hair on the right gastrocnemius muscle was subsequently removed. The arrangement of animal experiments is detailed in Figure 4, in which the bottom of the rectangular Plexiglas container was placed with a *p*-Xylene membrane. This allows the transmission and reception of ultrasound waves throughout the DD water, coupling gel, and the muscle tissue. The distance, 50.9 mm, between the transducer and the gastrocnemius muscle was adjusted, and that was subjective to the transducer’s focal length. During the experiment, a bolus of UCAs suspension was injected into the vein of the rat’s tail. For each measurement, a series of 600 images covering the process of UCAs perfusion was acquired. The size of each frame of image was 58 × 25 mm^2^, and that was composed of 250 A-lines signals at a 100 μm scanning interval. The time perfusion curve was estimated from the ROI of 3.5 × 6.9 mm^2^ within each frame of collected signals. The whole process of animal experiments was approved by the Ethical Committee for Animal Study (approval number: 108180), National Cheng Kung University, Tainan, Taiwan.

### 2.4. Perfusion Parameters Estimation

The perfusion parameters were estimated from the Nakagami statistical distribution of UCAs backscattered signals. The Nakagami distribution, *f*(*R*), of the backscattered envelope (*R*) is formulated [30,34] as follows:

(1) $$f\left(R\right)=\frac{2m^{m}R^{2m-1}}{\Gamma \left(m\right)\Omega^{m}}\cdot e^{\left(-\frac{m}{\Omega }R^{2}\right)}\cdot U\left(R\right)$$

where *Γ*(·) and *U*(·) represent, respectively, the gamma function and unit step function. Ω and *m* denote, respectively, the scaling parameter and Nakagami parameter, and which may be formulated as follows:

(2) $$\Omega =E\left(R^{2}\right)$$

and

(3) $$m=\frac{\left|E{\left(R^{2}\right)}^{2}\right|}{E{\left|R^{2}-E\left(R^{2}\right)\right|}^{2}}$$

in which *E*(·) is the statistical mean. The scaling parameter and Nakagami parameter correspond to the average power of the backscattered envelope and shape of statistical distribution, respectively. Furthermore, the WMC Nakagami imaging has been developed and validated to be able to better visualize the scattering properties in local tissues and to preserve the backscattered strengths within a certain size of the gated window [35]. The WMC Nakagami imaging, denoted as $M_{com}\left(x,y\right)$, may be implemented by summing and averaging all Nakagami images, $M\left(x,y\right)$, over different sliding windows ranging from 1 to N times of the pulse length, and which may be formulated as follows:

(4) $$M_{com}\left(x,y\right)=\frac{1}{N}\overset{N}{\underset{i=1}{\sum }}M_{i}\left(x,y\right)$$

In the present study, WMC Nakagami parameter ratio imaging was further developed and aimed to reduce the effect of tissue clutter on WMC Nakagami imaging for improving the reproducibility of UCAs perfusion measurements. The WMC Nakagami parameter ratio imaging, denoted as $M_{cr}\left(x,y\right)$, is formulated as follows:

(5) $$M_{cr}\left(x,y\right)=\frac{1}{N}\overset{N}{\underset{i=1}{\sum }}\frac{M_{{high}_{i}}\left(x,y\right)}{M_{{low}_{i}}\left(x,y\right)}-1$$

where $M_{{high}_{i}}\left(x,y\right)$ and $M_{{low}_{i}}\left(x,y\right)$ represent the acquired Nakagami images with respect to the incidence of a certain higher and lower acoustic pressures, respectively. Subsequently, TNRC may be estimated from a sequence of $M_{cr}\left(x,y\right)$ corresponding to UCAs perfusions. Those of $M_{com}$ and $M_{cr}$ within the ROI of WMC sliding windows were further averaged and denoted, respectively, as $\bar{m_{com}}$ and $\bar{mr_{com}}$. The pseudo-color for displaying the Nakagami imaging was designated to reflect the magnitude of both $\bar{m_{com}}$ and $\bar{mr_{com}}$ ranging from 0 to 0.5 and 0 to 0.04, respectively.

Perfusion parameters, as mentioned in the Introduction section, including AT, TTP, MAX, BFV, and AUC [10,13], for further assessing the muscle perfusion, were estimated from the perfusion curves of backscattered power-based TICs, as well as those of Nakagami-parameter-based TNCs and TNRCs. Detail descriptions about the perfusion parameters are given in Table 2. Furthermore, BFV been verified by multivessel model [36] is proportional to the MAX, with the relationship given as follows:

(6) $$\mathrm{BFV}=d\times k\times \frac{2}{3}\mathrm{MAX}$$

where *d* and *k* denote, respectively, the ultrasound beam width and the slope of perfusion curve over the perfusion time between AT and TTP. The variation of perfusion parameters was assessed by the relative standard deviation (RSD) [37] given as follows:

(7) $$\mathrm{RSD}=\frac{\sigma }{\mu }\times 100\%$$

where *μ* and *σ* represent, respectively, the mean and standard deviation of the corresponding perfusion parameters. The one-way ANOVA test was analyzed for assessing the significance, with the *p*-value smaller than 0.05, of results.

## 3. Results

### 3.1. Flow Phantom Experiments

**Figure 5:**
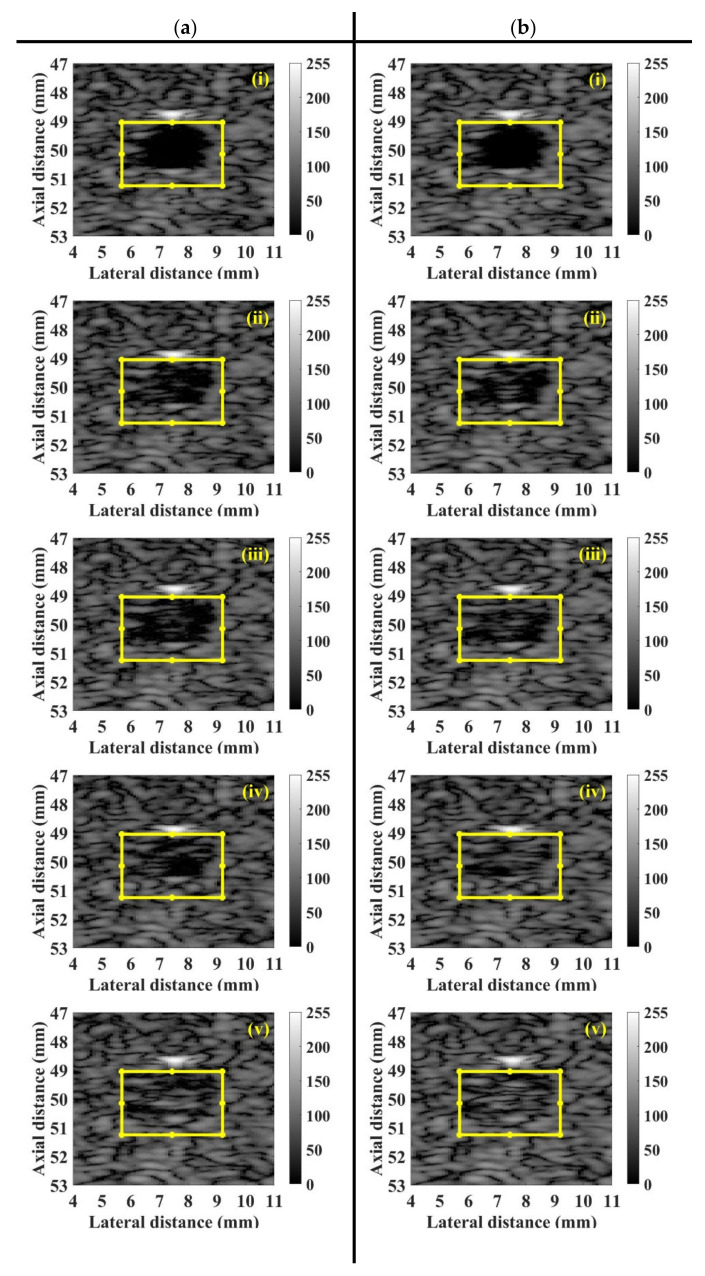
Typical B-mode images corresponding to the incidence of (**a**) 2.86 and (**b**) 4.37 MPa acoustic pressures into the flow phantom in which UCAs concentrations of (i) 0, (ii) 6.7, (iii) 13.3, (iv) 26.7, and (v) 53.3 × 106 UCAs/mL were administered.

**Figure 6:**
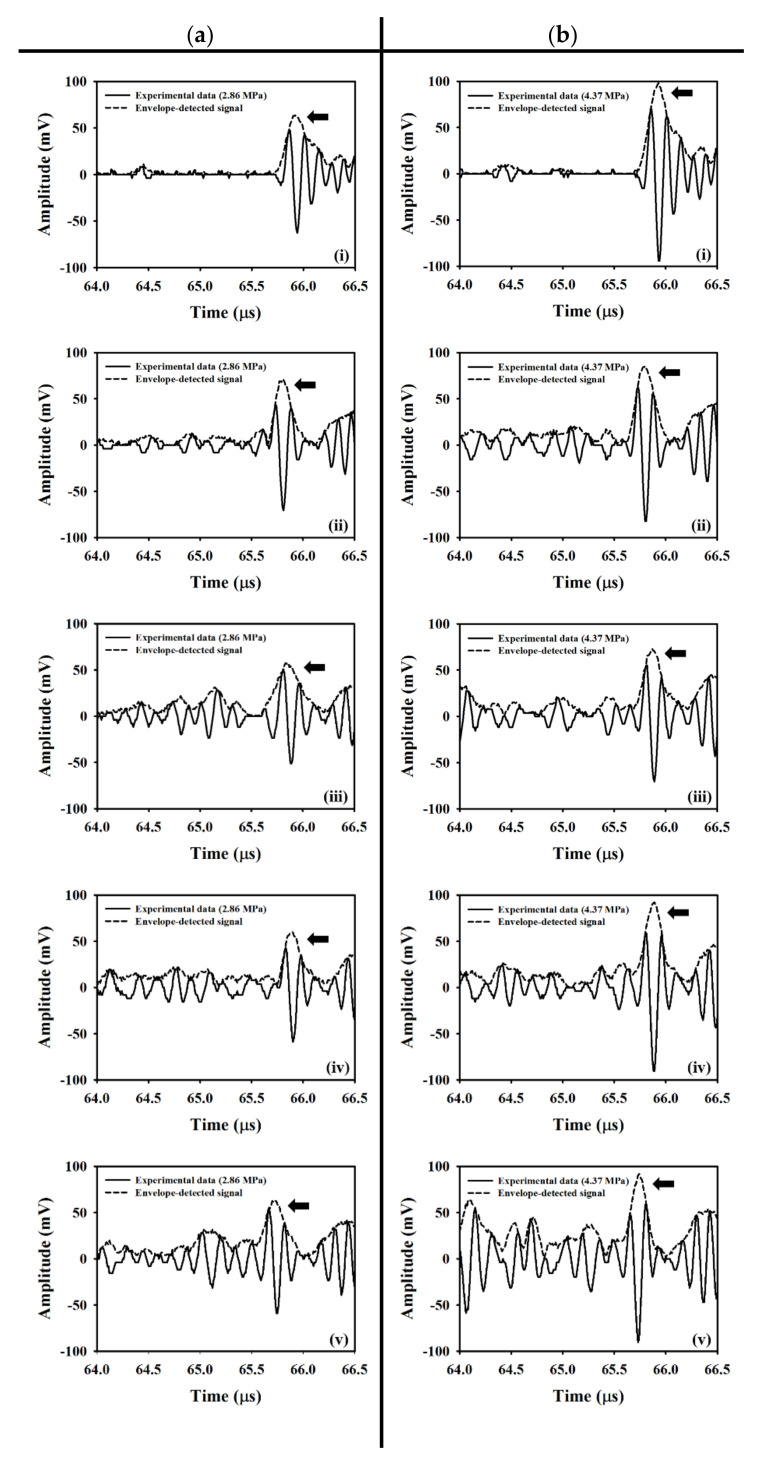
Typical radio-frequency signals and envelopes corresponding to the incidence of (**a**) 2.86 and (**b**) 4.37 MPa acoustic pressures into the flow phantom, where UCAs of various concentrations of (i) 0, (ii) 6.7, (iii) 13.3, (iv) 26.7, and (v) 53.3 × 10^6^ UCAs/mL were administered. The black arrow indicates the back wall of the flow conduit.

**Figure 7:**
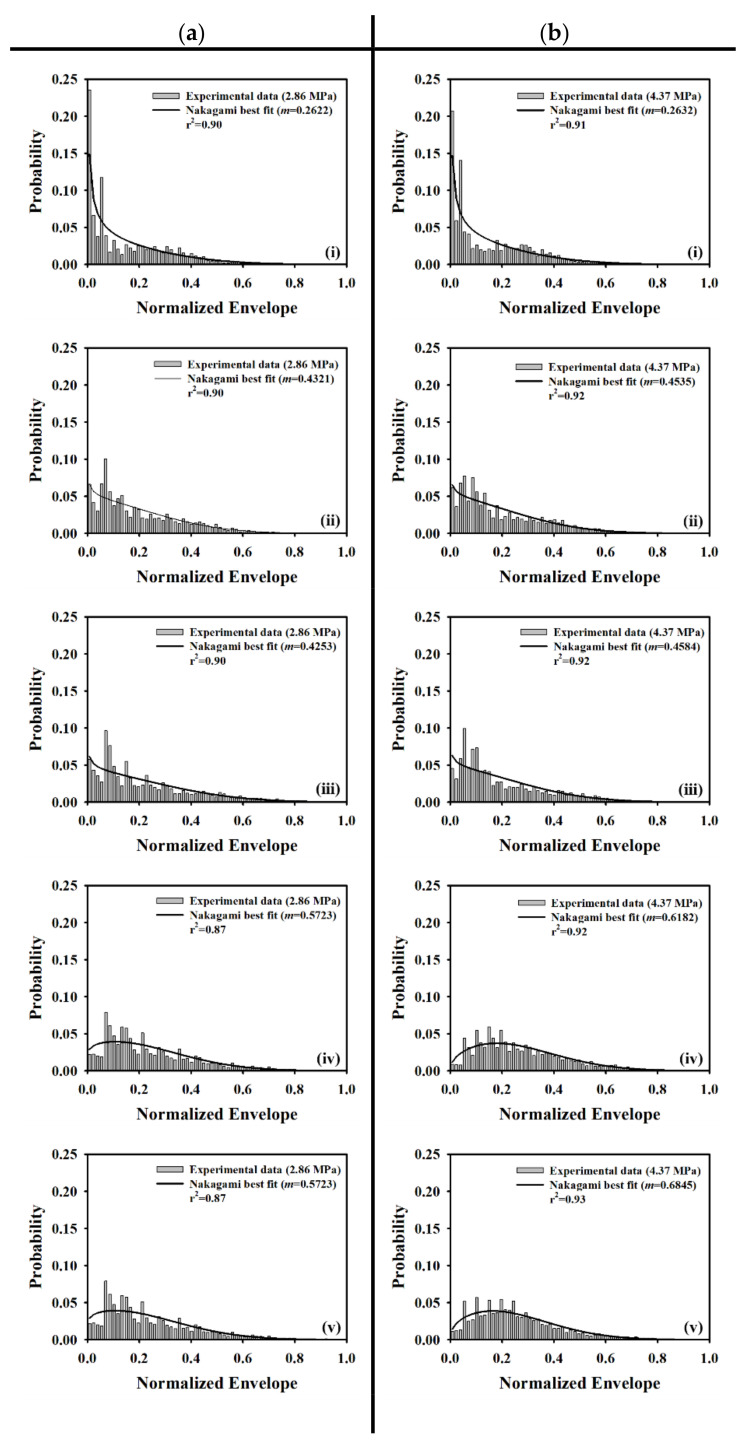
Typical probability density functions (PDFs) and fitted Nakagami distribution corresponding to the incidence of (**a**) 2.86 and (**b**) 4.37 MPa acoustic pressures into the flow phantom in which UCAs of various concentrations of (i) 0, (ii) 6.7, (iii) 13.3, (iv) 26.7, and (v) 53.3 × 10^6^ UCAs/mL were administered.

**Figure 8:**
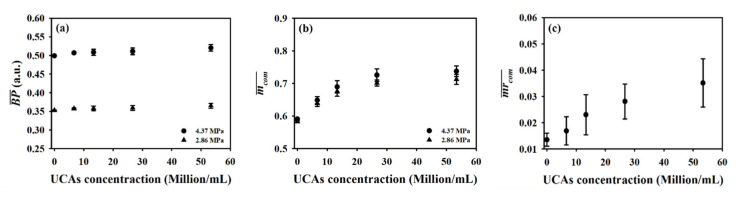
Average (**a**) backscattered power, (**b**) window-modulated compounding (WMC) Nakagami parameter, and (**c**) WMC Nakagami parameter ratio parameter as a function of UCAs concentration.

Figure 5a,b shows typical B-mode images corresponding to the incidence of 2.86 and 4.37 MPa acoustic pressures, respectively, into the flow phantom in which different UCAs concentrations of 0, 6.7, 13.3, 26.7, and 53.3 × 10^6^ UCAs/mL were administered. Apparently, the largest concentration of UCAs administration contributes to the highest echogenicity in the conduit area. The yellow rectangular area indicates the radio-frequency signals in the ROI of 2.5 × 4 mm^2^ for calculating the backscattered power, WMC Nakagami parameter, and WMC Nakagami parameter ratio parameter. The mean signal-to-noise ratios (SNR) corresponding to both incident acoustic pressures are higher than 22 dB and which satisfies the minimum SNR of 20 dB to estimate the Nakagami parameter for biological tissue characterization [38]. Moreover, the corresponding radio-frequency signals and envelopes acquired in the center stream of the flow conduit are given in Figure 6, in which the black arrow indicates the wall of the flow conduit. In response to the increase of incident acoustic pressures and UCAs concentrations, the strengths and variation of UCAs backscattered signals tended to increase accordingly. The corresponding PDFs and Nakagami parameters capable of describing the physical concentration, distribution, and properties of scatterers were given in Figure 7, in which the Nakagami parameters of backscattered envelopes generally increase with the increase of UCAs concentration and acoustic pressure. Furthermore, the average backscattered power ($\bar{BP})$, $\bar{m_{com}}$, and $\bar{mr_{com}}$ as a function of UCAs concentration are given in Figure 8, where these parameters apparently also tend to increase with the increase of UCAs concentrations. Specifically, the backscattered power of UCAs is highly affected by the incidence of acoustic pressure, as can be seen in Figure 8a; the $\bar{m_{com}}$, on the other hand, is less sensitive to that of the employed acoustic pressure. These results suggest that the Nakagami parameter is less sensitive to the backscattered power in response to different incident acoustic pressures, and thus that the blood perfusion in biological tissues may be better estimated by $\bar{mr_{com}}$, to reduce the influence of tissue clutters in biological tissues.

### 3.2. In Vivo Animal Experiments

**Figure 9:**
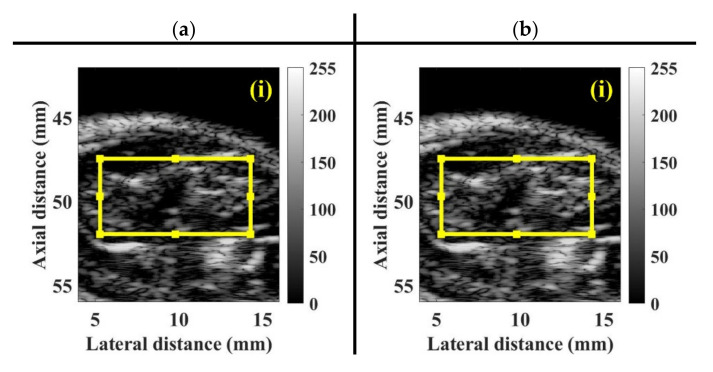

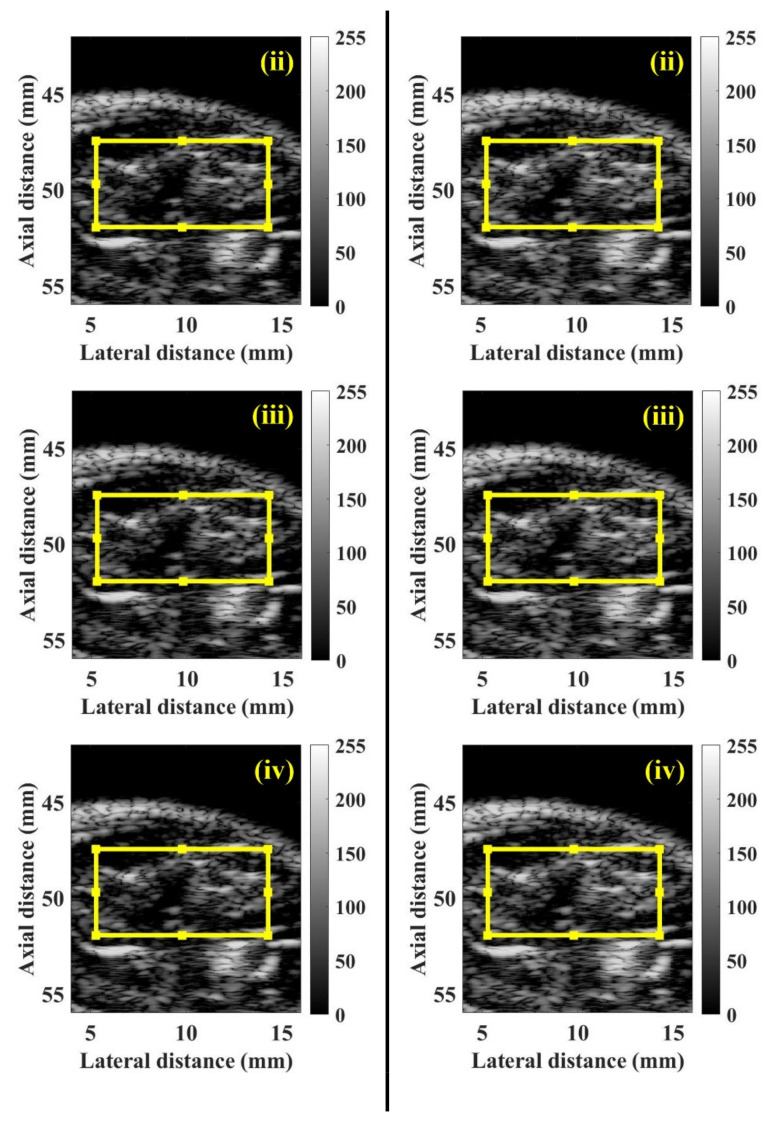
Typical B-mode images of muscle tissue at (i) pre-contrast, (ii) 55 s, (iii) 75 s, and (iv) 110 s, following UCAs administration corresponding to the incidence of (**a**) 2.86 and (**b**) 4.37 MPa acoustic pressures. The region of interest (ROI) is indicated by the yellow rectangular area.

**Figure 10:**
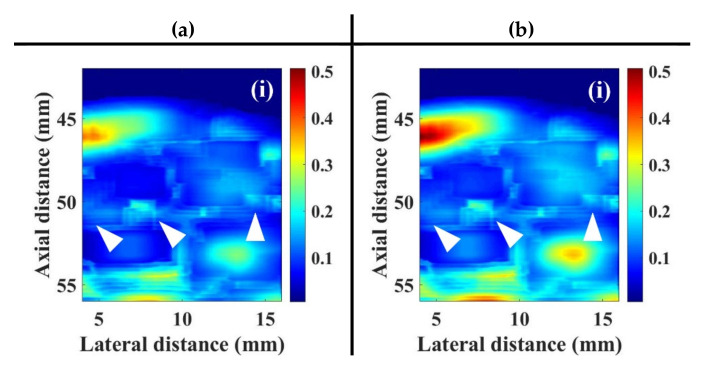

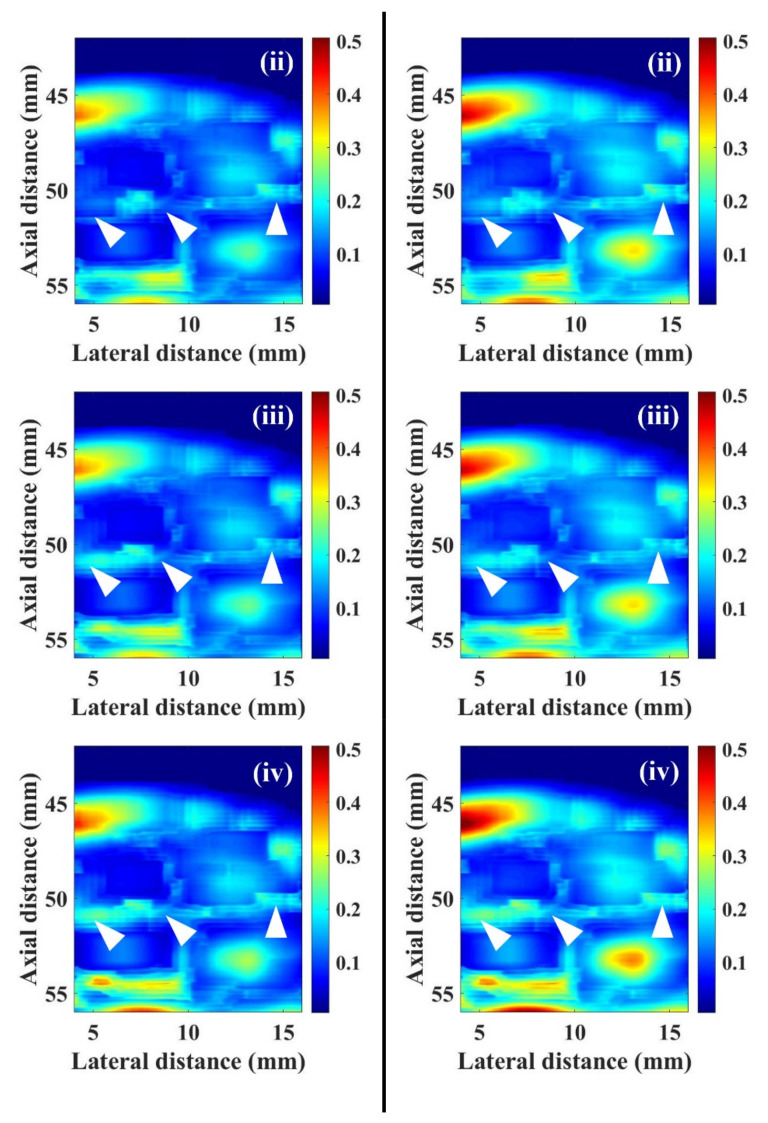
WMC Nakagami images of muscle tissue at (i) pre-contrast, (ii) 55 s, (iii) 75 s, and (iv) 110 s, following UCAs administration corresponding to the incidence of (**a**) 2.86 and (**b**) 4.37 MPa acoustic pressures. The perfusion areas of UCAs are indicated by white arrows.

For each animal experiment, a bolus of 10 μL UCAs suspensions with the concentration of 1.25 × 10^8^ UCAs/mL was manually injected into the tail vein of the rat, using a 26G syringe. A series of typical B-mode images, depicting the muscle tissue of pre-contrast, 55 s, 75 s, and 110 s, following UCAs administration associated with the incidence of (a) 2.86 and (b) 4.37 MPa acoustic pressures, was given in Figure 9. The ultrasonic signals of ROI achieved, respectively, the SNRs of 31.12 ± 0.06 dB and 32.39 ± 0.06 dB, with respect to the insonification of 2.86 and 4.37 MPa acoustic pressures. The B-mode images in the area of hindlimb GM apparently increased in response to the wash-in of UCAs at approximately 55 s, and then gradually decreased till that of the wash-out at approximately 110 s. The corresponding WMC Nakagami images, given in Figure 10, were implemented and that the Nakagami parameters were estimated from the sliding square windows, with the window lengths varying from one to eight times of the transducer pulse length. The pre-contrast WMC Nakagami images, as shown in Figure 10a(i),b(i), of UCAs perfusion regions depicted with dark-blue color and indicated by the white arrows, were resultant from the incidence of 2.86 and 4.37 MPa acoustic pressures, and which are with the mean $\bar{m_{com}}$ smaller than 0.15. The flow-in UCAs tended to increase the resultant mean $\bar{m_{com}}$ of the WMC Nakagami images in Figure 10a,b of (ii) 55 s, (iii) 75 s, and (iv)110 s to be larger than 0.2.

**Figure 11:**
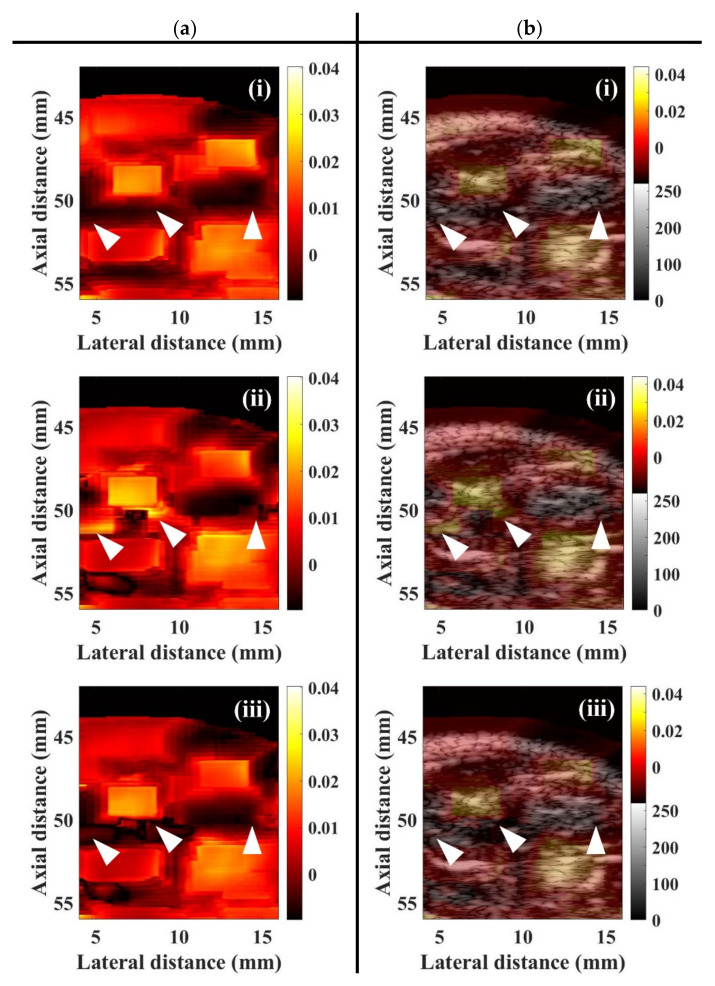

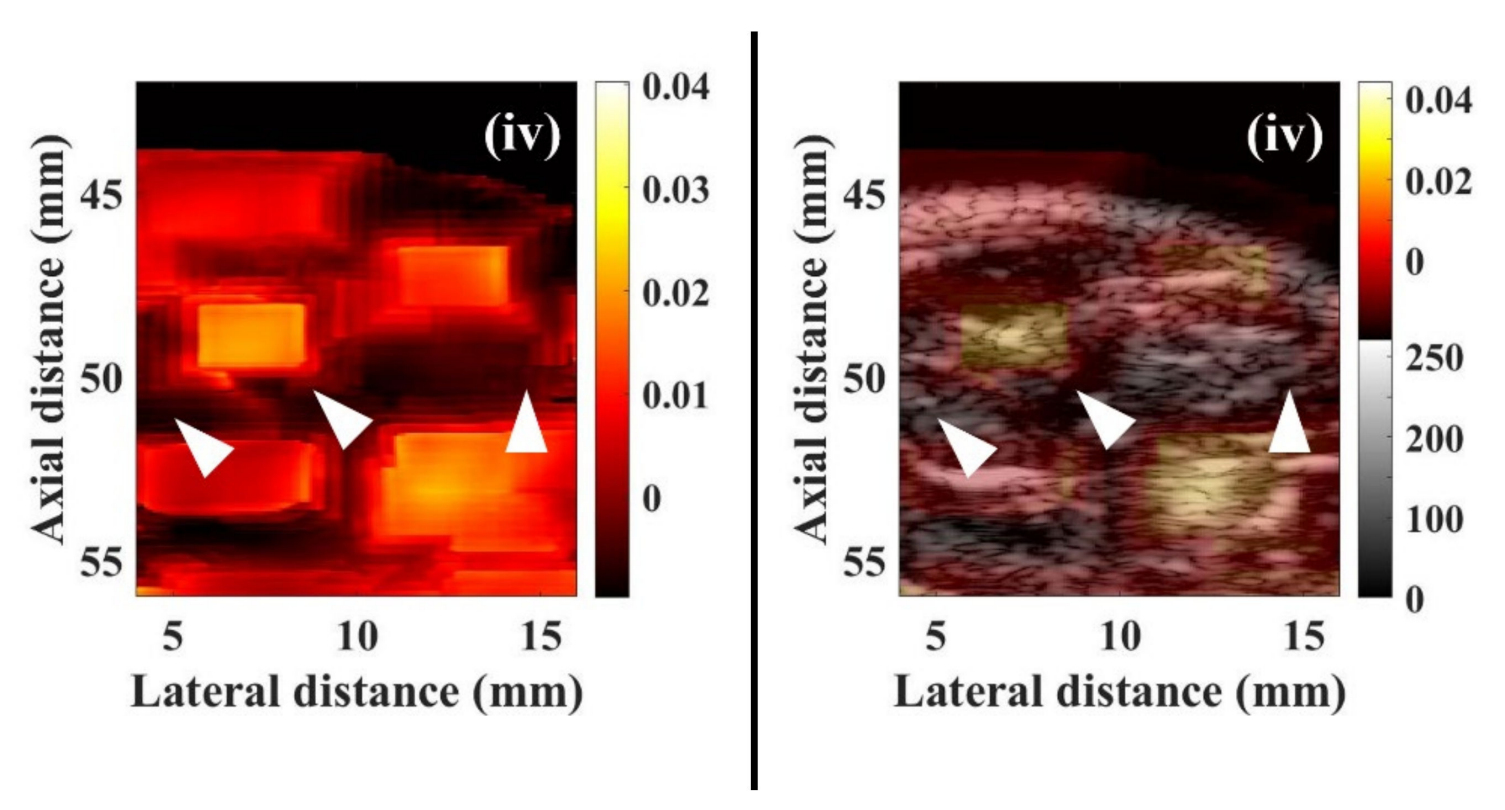
Typical (**a**) WMC Nakagami parameter ratio images and (**b**) fusion of WMC Nakagami parameter ratio and B-mode images at (i) pre-contrast, (ii) 55 s, (iii) 75 s, and (iv) 110 s, following UCAs administration. The areas of UCAs perfusion are indicated by white arrows.

In the present study, the WMC Nakagami parameter ratio images were implemented by taking the ratio of WMC Nakagami parameters corresponding to two different acoustic pressures, to reduce the effects of variations of scattering and subresolvable effect of UCAs, for better assessing muscle perfusion. Figure 11a is a series of typical WMC Nakagami parameter ratio images of muscle tissues, following the UCAs administration at (i) pre-contrast, (ii) 55 s, (iii) 75 s, and (iv) 110 s. The regions near the bottom of the lateral and medial GM muscle (as indicated white arrows) in the pre-contrast WMC Nakagami parameter ratio image may be discerned, as shown by the deep-red shadings in Figure 11a(i), with the $\bar{mr_{com}}$ smaller than 0.01. The perfusion areas (as indicated white arrows) following the first-pass of UCAs can also be readily discerned, as the bright-yellow color in Figure 11a(ii), with the mean $\bar{mr_{com}}$ larger than 0.025. The perfusion areas (as indicated white arrows) in Figure 11a(iii) become darker after the peak of the first pass of UCAs and that correspond to the mean $\bar{mr_{com}}$ larger than 0.015. The perfusion areas eventually fade to the deep-red shadings corresponding the flow-out of UCAs with the mean $\bar{mr_{com}}$ returning to values of smaller than 0.01 similarly to that of the pre-contrast. Furthermore, to better visualize and assess tissue perfusion, the image fused the WMC Nakagami parameter ratio images with the corresponding B-mode images was proposed and implemented. The fusion image provides not only the visualization of the anatomical structures by B-mode images, but also the additional Nakagami-parameter-based perfusion information with less subresolvable effect affected by tissue clutters. Figure 11b(i–iv) shows the corresponding WMC Nakagami parameter ratio/B-mode fusion images, in which the perfusion areas of UCAs are indicated as white arrows. Note that the fusion images provide abundant information about the anatomical tissue structure and areas of UCAs perfusion, and that the perfusion areas in the muscle tissues are with an appearance less blurring than those of WMC Nakagami and WMC Nakagami parameter ratio images.

**Figure 12:**
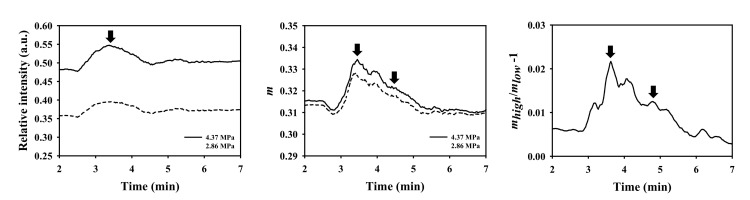
Typical (**a**) backscattered power, (**b**) WMC Nakagami parameter, and (**c**) WMC Nakagami parameter ratio as a function of time measured from the hindlimb GM of a rat. The black arrows indicated the peak concentrations corresponding to the first arrival and the first recirculation of administrated UCAs.

**Figure 13:**
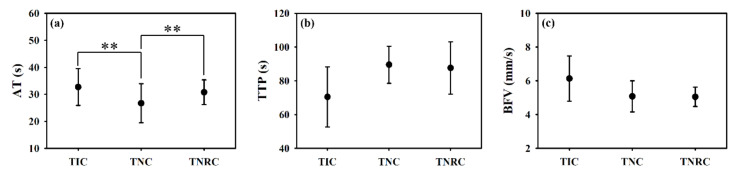
The comparison of (**a**) arrival time, (**b**) time-to-peak, and (**c**) blood flow velocity estimated from TIC, TNC, and TNRC (N = 20, **: *p* < 0.01, data were presented as mean ± standard deviation).

To further estimate the tissue perfusion by UCAs, the $\bar{BP}$, $\bar{m_{com}}$, and $\bar{mr_{com}}$ as a function of time, as given in Figure 12, were measured and then applied to estimate the perfusion parameters with respect to TIC, TNC, and TNRC, respectively. The TIC in Figure 12a clearly shows that backscattered strengths of signals resultant from the insonification of the higher acoustic pressure of 4.37 MPa are much larger than that of 2.86 MPa, and that the shapes and intervals of TIC remain consistent. The TIC tended to decrease gradually with the decrease of perfused UCAs concentration, and with that, the maximum $\bar{BP}$ corresponding to the first arrival of UCAs may be clearly discerned. It yet is difficult to discern the signals backscattered from the recirculated UCAs, as they were mixing with backscattering signals of tissue clutters, as well as affected by the incidence of acoustic pressures, transducer beam characteristics, imaging system factors, and tissue properties. On the other hand, the TNC in Figure 12b tends to be able to preserve the first recirculation of UCAs, as indicated by the arrows; however, as a result of the subresolvable effect [32], the second recirculation of UCAs was barely observable. Results of Figure 12c demonstrated that the TNRC approach in comparison with those of TIC and TNC approaches is able to better reduce the subresolvable effect and to preserve detailed information about the UCAs perfusion in the tissue. Furthermore, the corresponding perfusion parameters, including AT, TTP, and BFV in Figure 13, estimated from TNRC, are generally in good agreement with those of from TNC and TNRC. The average AT (26.73 s) estimated from TNC was generally much shorter than those of calculated by TIC (32.54 s, *p* < 0.001) and TNRC (30.81 s, *p* < 0.001), as shown in Figure 13a. This could be partially due to a large variation of backscattering signals around the onset flow-in UCAs corresponding to the largest RSD of AT estimated from TNC, as given in Table 3. In particular, the AT and BFV parameters derived from the TNRC approach achieved a smaller RSD than those from the TIC and TNC approaches.

## 4. Discussion

It is of importance to efficiently reduce the effects of tissue clutters to allow CEUS imaging to be able to better visualize and assess the perfusion of a local tissue for in situ diagnosing the muscular diseases. The variation of backscattering signals of UCAs and such ultrasound system factors as gain, time-gain compensation, and dynamic range are also primary factors to affect the accuracy of perfusion measurement by conventional TIC approach. These factors have been found to be able to be eliminated by the statistical analysis of signals, using Nakagami statistics [30,31]. Furthermore, by replacing the ultrasound intensity with the Nakagami statistical parameter, the TNC approach was found capable of better tolerating tissue clutters and UCAs attenuations, without the need to utilize an additional wall filter for estimating the flow from phantom experiments [32,33]. The accuracy of the TNC approach on the flow estimation was improved because this approach is less affected by UCAs concentrations, incident acoustic pressures, attenuation, and intrinsic noise of the system [32]. Nevertheless, the reproducibility and robustness of the TNC approach for assessing tissue perfusion could still be affected by a variety of small vessels, hydrostatic pressures, and heterogeneous tissue clutters [39,40]. These studies utilized a 25 MHz ultrasound in an attempt to increase the spatial resolution of CEUS images. The incident frequency, however, is far from the resonance frequency of the UCAs [31]. The mismatch between the employed ultrasound frequency and the resonance frequency of UCAs tended to limit a thorough study on the pressure-dependent behavior of UCAs’ scattering properties [41,42]. The abovementioned issues were further alleviated by the utilization of WMC Nakagami parameter ratio approach with CEUS imaging in the present study. Specifically, the WMC Nakagami parameter ratio image is capable of preserving the smoothness of parametric image and reducing the tissue clutters effect by utilizing both the window-modulated compounding imaging and the ratio of Nakagami parameters resultant from the acquired signals associated with the incidence of two different acoustic pressures. Consequently, it allows the Nakagami-parameter-based CEUS imaging to be a suitable candidate approach for better assessing the tissue perfusion than that of conventional ultrasound-intensity-based CEUS imaging. Moreover, the spatial resolution of the Nakagami image typically is degraded and insufficient to assess and characterize the properties of a local tissue in detail than that of B-mode image due to the use of a minimum window size of three times of the transducer pulse length for Nakagami imaging [14,43]. This issue may be further resolved by the proposed fusion imaging, using ultrasound B-mode and WMC Nakagami parameter ratio images. The fusion imaging provides the information of not only the tissue structures in the perfusion area and surroundings, but also their scattering and perfusion properties.

In general, the estimations of perfusion parameters in flow phantom experiments by the TNRC approach are consistent with those of the TIC and TNC approaches, as well as those reported in previous studies [32,33]. The subsequent animal experiments resulted in a slight increase of echogenicity of B-mode images between the pre-contrast and post-contrast of UCAs, and that however is short of sensitivity to detail the variations of echogenicity between the wash-in and wash-out of UCAs. The estimation of perfusion parameters by using TIC approach was easily contaminated by the clutter signals from the surrounding tissues and vessel walls. The variation of perfusion parameters in animal experiments also tended to be higher, owing to the fact that slight movement of the animal was occasionally found during data acquisition in experiments. The WMC Nakagami images are generally less affected by the tissue clutter to discern the area of tissue images with or without the perfusion of UCAs than that of the B-mode images. However, although the SNRs of ultrasound signals of both flow phantom and animal experiments were higher than 20 dB, the resultant WMC Nakagami images appeared to have blurred features and reduced spatial resolution. This is partially due to the subresolvable effect that may reduce the dynamic range of Nakagami imaging to be between 0.24 and 0.36, as reported in previous studies [32]. The subresolvable effect on WMC Nakagami images may be readily discerned in Figure 10, in which it led the images at 75 and 110 s post-contrast of UCAs, as the indicated areas with bright-blue color near the bottom of the lateral and medial GM muscles, to be with the blurred appearance and reduced spatial resolution. The predominant cause of this result is due to the variation of backscattered statistics associated with the fascia of GM muscle.

The WMC Nakagami parameter ratio imaging and TNRC approach in the present study have further demonstrated themselves to be able to better describe and assess the perfusion of UCAs in the muscle, by reducing the subresolvable effect of tissue clutters with the normalization of WMC Nakagami parameters corresponding to the incidence of two acoustic pressures alternatively. The perfusion parameters estimated from the newly developed WMC Nakagami parameter ratio and TNRC approach were generally comparable to those of the TIC and TNC approaches. Specifically, the AT (26.73 s) estimated from the TNC approach is significantly shorter than that of the TIC (32.54 s) and TNRC (30.81 s) approaches, and that may readily correspond to the dramatic decrease of WMC Nakagami parameters to the onset flow-in of UCAs in Figure 12b. The RSD of AT and BFV estimated from the TNRC approach is smaller than that of the TIC and TNC approaches. This indicates that the TNRC approach has better tolerance on speckle noise and tissue clutters for reducing the subresolvable effect. The WMC Nakagami parameter ratio imaging is not yet suitable to be adopted for assessing the non-perfusion areas with CEUS imaging, since the surrounding tissues tend to be more rigid and exhibit less pressure-dependent behavior than those of UCAs [17,18]. Apparently, this issue may be alleviated by the introduced WMC Nakagami parameter ratio/B-mode fusion image to preserve both the anatomical structure and perfusion information. Consequently, it is worthwhile to further design and develop a dedicated compound ultrasound system to combine the CEUS images and perfusion parameters into a single metric for comprehensively diagnosing tissue perfusion. The metric containing certain pathological meanings may also be served as inputs for automatically classifying the pathological tissue by machine- and deep-learning classifiers in a future study.

## 5. Conclusions

This study employed phantom measurements and in vivo experiments on the rat leg muscles, to assess the UCAs perfusion, utilizing the newly developed WMC Nakagami parameter ratio imaging and TNRC approach. The animal experiments achieved some important findings and practical considerations, given as follows. Firstly, the subresolvable effect leads the WMC Nakagami imaging to have a blurred appearance and reduced spatial resolution, and that can be readily discerned near the bottom of the lateral and medial GM muscle. It thus can further reduce the reproducibility and robustness of the TNC approach for assessing tissue perfusion. Secondly, the AT of 26.73 s derived from the TNC approach is much shorter than those of TIC (32.54 s) and TNRC (30.81 s), and that corresponds to a dramatic decrease of WMC Nakagami parameters to the onset flow-in of UCAs. Thirdly, the RSDs of both AT and BFV derived by the newly developed TNRC approach are smaller than those of TIC and TNC approaches, indicating that the WMC Nakagami parameter ratio imaging may effectively suppress the subresolvable effect. On the other hand, the WMC Nakagami parameter ratio imaging is not suitable to assess the non-perfusion areas from tissues, owing to their limited pressure-dependent behavior. This issue was alleviated by fusing the newly developed WMC Nakagami parameter ratio and B-mode image to preserve both the anatomical structure and perfusion information. Furthermore, various parameters and CEUS images corresponding to a certain pathological meaning are worthwhile to be introduced into machine- and deep-learning classifiers for automatically classifying and diagnosing the perfusion of tissues.

## Figures and Tables

**Table 1:** Characteristics of the applied transducer.

Center frequency	7.3 MHz
−6 dB bandwidth	3.2 MHz
*f*-number	2.8
Depth of focus	53.3 mm
Aperture size	19 mm

**Table 2:** Perfusion parameters derived from the perfusion curve.

Perfusion Parameter	Descriptions
Arrival time (AT)	Time at the arrival of UCAs
Peak value (MAX)	Peak value of the perfusion curve corresponding to the flow-in UCAs
Time-to-peak (TTP)	Interval between AT and the time of MAX
Blood flow velocity (BFV)	Mean blood flow velocity within the duration of UCAs perfusion
Area under perfusion curve (AUC)	Area under the curve of UCAs perfusion

**Table 3:** Relative standard deviation (RSD) of perfusion parameters estimated from different time curves of contrast-enhanced ultrasound (CEUS) imaging.

	Method	TIC	TNC	TNRC
Parameter				
AT		19.27%	53.78%	10.07%
TTP		25.20%	12.29%	17.12%
MAX		33.33%	11.11%	26.08%
BFV		19.59%	14.93%	9.24%
AUC		24.04%	8.62%	17.48%

## References

[1] Marini M., Veicsteinas A. (2010). The exercised skeletal muscle: A review. Eur. J. Transl. Myol..

[2] Aspelin P., Ekberg O., Thorsson O., Wilhelmsson M., Westlin N. (1992). Ultrasound examination of soft tissue injury of the lower limb in athletes. Am. J. Sports Med..

[3] Draghi F., Zacchino M., Canepari M., Nucci P., Alessandrino F. (2013). Muscle injuries: Ultrasound evaluation in the acute phase. J. Med. Ultrasound.

[4] Lovitt S., Marden F.A., Gundogdu B., Ostrowski M.L. (2004). MRI in myopathy. Neurol. Clin..

[5] Matin P., Lang G., Carretta R., Simon G. (1983). Scintigraphic evaluation of muscle damage following extreme exercise: Concise communication. J. Nucl..

[6] Ament W., Lubbers J., Rakhorst G., Vaalburg W., Verkerke G.J., Paans A.M., Willemsen A.T. (1998). Skeletal muscle perfusion measured by positron emission tomography during exercise. Pflügers Arch..

[7] Nuutila P., Kalliokoski K. (2000). Use of positron emission tomography in the assessment of skeletal muscle and tendon metabolism and perfusion. Scand. J. Med. Sci. Sports.

[8] Garcia J. (2000). MRI in inflammatory myopathies. Skeletal Radiol..

[9] Lutz A.M., Weishaupt D., Amann-Vesti B.R., Pfammatter T., Goepfert K., Marincek B., Nanz D. (2004). Assessment of skeletal muscle perfusion by contrast medium first-pass magnetic resonance imaging: Technical feasibility and preliminary experience in healthy volunteers. J. Magn. Reson. Imaging.

[10] Weber M.A., Krix M., Delorme S. (2007). Quantitative evaluation of muscle perfusion with CEUS and with MR. Eur. Radiol..

[11] Peetrons P. (2002). Ultrasound of muscles. Eur. Radiol..

[12] Lin Y.-H., Hsieh M.-Y., Su F.-C., Wang S.-H. (2014). Assessment of the kinetic trajectory of the median nerve in the wrist by high-frequency ultrasound. Sensors.

[13] Krix M., Weber M.A., Krakowski-Roosen H., Huttner H.B., Delorme S., Kauczor H.U., Hildebrandt W. (2005). Assessment of skeletal muscle perfusion using contrast-enhanced ultrasonography. J. Med. Ultrasound.

[14] Lin Y.H., Huang C.C., Wang S.H. (2011). Quantitative assessments of burn degree by high-frequency ultrasonic backscattering and statistical model. Phys. Med. Biol..

[15] Maruvada S., Shung K.K., Wang S.-H. (2002). High-frequency backscatter and attenuation measurements of porcine erythrocyte suspensions between 30–90 MHz. Ultrasound Med. Biol..

[16] Shung K.K. (2006). Diagnostic Ultrasound: Imaging and Blood Flow Measurements.

[17] Cosgrove D. (2006). Ultrasound contrast agents: An overview. Eur. J. Radiol..

[18] Sboros V. (2008). Response of contrast agents to ultrasound. Adv. Drug. Deliv. Rev..

[19] Huang C.C., Lin Y.H., Wang S.H. (2009). The Effect of Kinetic Properties on Statistical Variations of Ultrasound Signals Backscattered from Flowing Blood. Jpn. J. Appl. Phys..

[20] Kaul S. (2008). Myocardial contrast echocardiography: A 25-year retrospective. Circulation.

[21] Siracusano S., Bertolotto M., Ciciliato S., Valentino M., Liguori G., Visalli F. (2011). The current role of contrast-enhanced ultrasound (CEUS) imaging in the evaluation of renal pathology. World J. Urol..

[22] Duerschmied D., Olson L., Olschewski M., Rossknecht A., Freund G., Bode C., Hehrlein C. (2006). Contrast ultrasound perfusion imaging of lower extremities in peripheral arterial disease: A novel diagnostic method. Eur. Heart J..

[23] Duerschmied D., Maletzki P., Freund G., Olschewski M., Seufert J., Bode C., Hehrlein C. (2008). Analysis of muscle microcirculation in advanced diabetes mellitus by contrast enhanced ultrasound. Diabetes Res. Clin. Pract..

[24] Thomas K.N., Cotter J.D., Lucas S.J., Hill B.G., van Rij A.M. (2015). Reliability of contrast-enhanced ultrasound for the assessment of muscle perfusion in health and peripheral arterial disease. Ultrasound Med. Biol..

[25] Hotfiel T., Heiss R., Swoboda B., Kellermann M., Gelse K., Grim C., Strobel D., Wildner D. (2018). Contrast-enhanced ultrasound as a new investigative tool in diagnostic imaging of muscle injuries—A pilot study evaluating conventional ultrasound, CEUS, and findings in MRI. Clin. J. Sport Med..

[26] Krix M., Krakowski-Roosen H., Kauczor H.U., Delorme S., Weber M.A. (2009). Real-time contrast-enhanced ultrasound for the assessment of perfusion dynamics in skeletal muscle. Ultrasound Med. Biol..

[27] Song Y., Li Y., Wang P.J., Gao Y. (2014). Contrast-enhanced ultrasonography of skeletal muscles for type 2 diabetes mellitus patients with microvascular complications. Int. J. Clin. Exp. Med..

[28] Forsberg F., Merton D., Liu J., Needleman L., Goldberg B. (1998). Clinical applications of ultrasound contrast agents. Ultrasonics.

[29] Tao Q., Wang Y., Fish P., Wang W., Cardoso J. (2004). The wall signal removal in Doppler ultrasound systems based on recursive PCA. Ultrasound Med. Biol..

[30] Tsui P.-H., Wang S.-H. (2004). The effect of transducer characteristics on the estimation of Nakagami parameter as a function of scatterer concentration. Ultrasound Med. Biol..

[31] Tsui P.H., Chang C.C. (2007). Imaging local scatterer concentrations by the Nakagami statistical model. Ultrasound Med. Biol..

[32] Tsui P.H., Yeh C.K., Chang C.C. (2009). Microvascular Flow Estimation by Microbubble-Assisted Nakagami Imaging. Ultrasound Med. Biol..

[33] Gu X., Wei M., Zong Y., Jiang H., Wan M. (2013). Flow quantification with nakagami parametric imaging for suppressing contrast microbubbles attenuation. Ultrasound Med. Biol..

[34] Shankar P.M. (2001). Ultrasonic tissue characterization using a generalized Nakagami model. IEEE Trans. Ultrason. Ferroelectr. Freq. Control.

[35] Tsui P.-H., Ma H.-Y., Zhou Z., Ho M.-C., Lee Y.-H. (2014). Window-modulated compounding Nakagami imaging for ultrasound tissue characterization. Ultrasonics.

[36] Krix M., Kiessling F., Farhan N., Schmidt K., Hoffend J., Delorme S. (2003). A multivessel model describing replenishment kinetics of ultrasound contrast agent for quantification of tissue perfusion. Ultrasound Med. Biol..

[37] Everitt B.S., Skrondal A. (2010). The Cambridge Dictionary of Statistics.

[38] Tsui P.-H., Wang S.-H., Huang C.-C., Chiu C.-Y. (2005). Quantitative Analysis of Noise Influence on the Detection of Scatterer Concentration by Nakagami Parameter. J. Med. Biol. Eng..

[39] Sassaroli E., Hynynen K. (2005). Resonance frequency of microbubbles in small blood vessels: A numerical study. Phys. Med. Biol..

[40] Dave J.K., Halldorsdottir V.G., Eisenbrey J.R., Liu J.B., McDonald M.E., Dickie K., Leung C., Forsberg F. (2011). Noninvasive Estimation of Dynamic Pressures In Vitro and In Vivo Using the Subharmonic Response From Microbubbles. IEEE Trans. Ultrason. Ferroelectr. Freq. Control.

[41] Chen Q., Zagzebski J., Wilson T., Stiles T. (2002). Pressure-dependent attenuation in ultrasound contrast agents. Ultrasound Med. Biol..

[42] Sboros V., MacDonald C.A., Pye S.D., Moran C.M., Gomatam J., McDicken W.N. (2002). The dependence of ultrasound contrast agents backscatter on acoustic pressure: Theory versus experiment. Ultrasonics.

[43] Ho M.-C., Lin J.-J., Shu Y.-C., Chen C.-N., Chang K.-J., Chang C.-C., Tsui P.-H. (2012). Using ultrasound Nakagami imaging to assess liver fibrosis in rats. Ultrasonics.

